# Doxycycline-encapsulated solid lipid nanoparticles for the enhanced antibacterial potential to treat the chronic brucellosis and preventing its relapse: in vivo study

**DOI:** 10.1186/s12941-019-0333-x

**Published:** 2019-11-09

**Authors:** Seyed Mostafa Hosseini, Abbas Farmany, Roghayyeh Abbasalipourkabir, Sara Soleimani Asl, Alireza Nourian, Mohammad Reza Arabestani

**Affiliations:** 1 Department of Microbiology, Faculty of Medicine, Hamadan University of Medical Sciences, Hamadan, Iran; 2 Dental Research Center, School of Dentistry, Hamadan University of Medical Sciences, Hamadan, Iran; 3 Department of Biochemistry, Faculty of Medicine, Hamadan University of Medical Sciences, Hamadan, Iran; 4 Department of Anatomical Sciences, Faculty of Medicine, Hamadan University of Medical Sciences, Hamadan, Iran; 5 Department of Pathobiology, School of Veterinary Science, Bu-Ali Sina University, Hamedan, Iran; 6 Brucellosis Research Center, Hamadan University of Medical Sciences, Hamadan, Iran

**Keywords:** *Brucella melitensis*, Doxycycline-encapsulated solid lipid nanoparticles, In vivo, Spleen, Liver, Relapse

## Abstract

**Background:**

Brucellosis is one of the most important infection of diseases. Due to its large period of treatment and survival ability of bacteria inside the macrophages, relapse of this disease is the main challenge, especially, after the treatment.

**Objective:**

The current study was carried out to evaluate the antibacterial effect of solid lipid nanoparticles loaded with doxycycline on the *Brucella melitensis* in in vivo conditions.

**Methods:**

The double emulsion synthesized doxycycline-encapsulated solid lipid nanoparticles (DOX-SLN) was characterized using DLS and FE-SEM. The efficacy of the DOX-SLN on the acute and chronic Wistar rat infected brucellosis was investigated. The pathological assessments were made on the spleen and liver in the treated rates.

**Results:**

The in vivo experimental results demonstrated that the treated rats with DOX-SLN had significantly decreased the *B. melitensis* CFUs in their spleen and liver compared to that of the treated rates with free doxycycline and untreated ones. The pathologic results indicate that the improvement trend of spleen and liver tissues in rats treated by DOX-SLN was satisfactory.

**Conclusion:**

According to in vivo results, the DOX-SLN has better effects on the treatment of chronic brucellosis. Therefore, DOX-SLN is recommended to treat the brucellosis and avoid its relapse. 
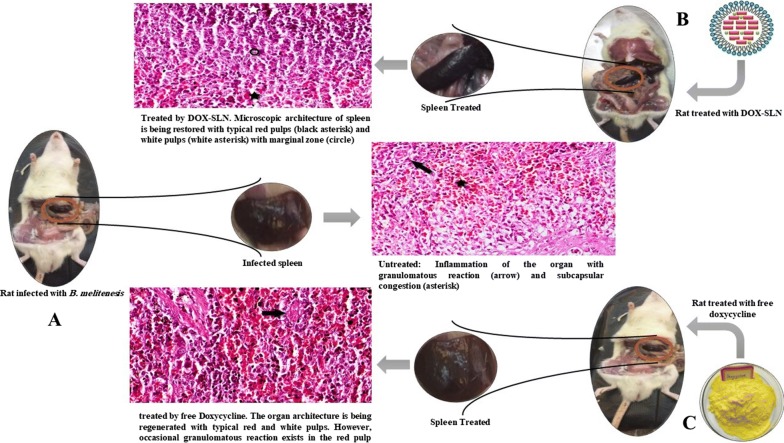

## Introduction

Brucellosis is a bacterial disease common between humans and animals that is transmitted by direct or indirect human contact with infected animals or infected dairy products. Clinical manifestations in brucellosis range from asymptomatic infections to acute, sub-acute and chronic forms. It is a systemic infection that can affect any organ or tissue in the body that can be manifested by nonspecific symptoms such as fever, bone pain, muscle aches, weakness, sweating, weight loss, and headaches. Arthritis is the most common complication of this disease [1]. Brucellosis is endemic in Iran and the Middle East, according to research, and the most common cause is *Brucella melitensis* [2]. *Brucella* is an intracellular bacterium that plays a key role in the eradication of the disease in humans by phagocytosis of polymorphonuclear and mononuclear cells including macrophages [3]. Following *Brucella* entry into the body by macrophages، monocytes and neutrophils, about 90% of them are killed within the first few hours, some of which live inside macrophages and find a place to proliferate within the cell [1, 4, 5].

The World Health Organization recommends the simultaneous use of gentamicin and doxycycline in order for preventing the relapse of brucellosis [6]. However, as gentamicin is nephrotoxic, the simultaneous use of streptomycin and doxycycline is preferred. Nowadays, routine ways for treating brucellosis include the simultaneous use of doxycycline and rifampicin for 6 weeks, the simultaneous use of doxycycline and streptomycin for 2–3 weeks, and the simultaneous use of doxycycline and gentamicin for 1 week. Furthermore, the simultaneous oral use of doxycycline and rifampicin for 45 days has been become popular among patients because it has no adverse side effect and the use is also easy. However, this treatment is less effective, particularly for complex forms of the disease (failure rate of about 15%) [7].

In spite of the notable number of new antibiotics, treatment of intracellular pathogens is still a great challenge [8]. To date, no antibiotic treatment has been reported to annihilate *Brucella* intracellular infections. Designing and developing a carrier system for drugs to be effectively endocytosed by phagocytic cells and release drugs contained within is the main challenge in intracellular chemotherapy. Targeted drug delivery system can be considered a proper tool to overcome such problems. Nowadays, pharmaceutical nanoparticles have many unique characteristics which can promote their efficacy in many aspects. Their precise formulation would enhance their stability and facilitate their dissolution for reaching the biological levels, which accelerate the treatment process. Moreover, it has been demonstrated that developing new medicines by itself is not enough for treating diseases. The low solubility of some medicines in water and low bioavailability of their molecules are fundamental problems in this regard. Therefore, there is an urgent need for developing new drug delivery systems for surmounting these obstacles. Apparently, the new systems to be developed should have some basic characteristics; it must be nontoxic, has a sufficient drug capacity, and also has the ability to carry and deliver the drug at the targeted places in the systematic and controllable way [9]. These carrier systems should have enough capacity for drug loading, being non-toxic and able to release drug slowly and steady. Solid lipid nanoparticles are a good candidates to drug delivery. These nanoparticles have been used for drug encapsulation [10, 11].

Although having some limitations, SLNs benefit from important properties. For example, SLNs would protect the drugs from enzymatic and chemical decompositions. As there is no need to use organic solvents, the production of SLNs is easier than other types of drugs [12, 13]. Accordingly, the present study was set to develop a novel method for treating brucellosis in order to minimize the use of conventional antibiotics, and to evaluate the therapeutic efficacy of solid lipid nanoparticles loaded with doxycycline in the treatment of acute and chronic brucellosis infections.

## Materials and methods

### Materials

Doxycycline Hyclate, Sorbitanmonooleate, polioxiethylene-20-sorbitan monooleate, stearic acid, Poloxamer407 and Tween 80 were purchased from Sigma-Aldrich (USA). Palm oil (hydrogenated) was purchased from Condea (Germany). Double distilled water was used throughout the experiments. *B. melitensis M16* was purchased from Razi Vaccine and Serum Research Institute, Iran. J774A.1 cell line was obtained from Pasteur Institute, Iran.

### Synthesis and characterization of NPs

The synthesis and characterization of SLN were previously reported by our group [14]. Double emulsion/melt dispersion method was used for the nanoparticle synthesis. In brief, the primary emulsion consisted of 0.6 g palm oil (Witten, Germany), 0.5 ml distilled water, 30 mg Doxycycline (Sigma Aldrich, USA), and 60 mg poloxamer (Sigma Aldrich, USA). These compounds were well homogenized using a sunicator at 60 °C (Skyman, China). Next, in order to prepare the secondary emulsion, the primary emulsion was added to 30 mg Tween-80 and mixed using ultrasonic device (Bandelin sonopls, Berlin, Germany) at 45% amplitude (20w) at a pulse rate of 10 s on and 5 s off from 1 min. Lastly, it slowly added to 30 ml 5 °C distilled water. For long term storage, the compound were lyophilized. The prepared nanoparticles were characterized by FTIR, DSC, Particle size, Zeta potential, Polydispersity Index (PDI) and FE-SEM. The doxycycline content of DOX-SLN was monitored by an HPLC instrumentation equipped with a UV detector. The release of DOX-SLN was evaluated using a dialysis bag (cut-off 12,000, Dialysis tubing, Sigma Chem. Co., Missouri, USA). MTT assay was applied to evaluate the cytotoxicity of synthesized nanoparticles [15, 16].

### Animal experiment: in vivo infection treatment assay

Male Wistar rats with an age range from 6 to 8 weeks and weight of 250(± 30) g were purchased from Hamadan University of medical sciences.

Rats were infected by intraperitoneally injection of 1.5 × 10^6^ CFUs of *B. melitensis*. After 10 days of infection (acute stage of infection) and 6 weeks of infection (chronic stage of infection), the rats were divided into four groups.

All experiments involving animals were performed according to the guidelines for maintenance, surveillance and usage of laboratory animals published by the National Institute of Health United State (NIH publication No. 85-23, revised 1985). Moreover, the study was approved by the ethics committee of the Hamadan University of Medical Sciences (No: IRUMSHA. REC. 1395066).

The acute phase was conducted as following; the rats were intraperitoneally exposed to *B. melitensis* for 10 days, one group of rats was kept untreated as controls and the other three groups (five rats per groups) were treated with three different doses of (1) free doxycycline (2.5 mg/kg), (2) DOX-SLN (2.5 mg/kg) and (3) free SLN (no antibiotics) at days 11, 13 and 15 postinfection.

The chronic phase was conducted as following; the rats were intraperitoneally exposed to *B. melitensis* for 5 weeks, no treatment was conducted on one group and the rats in this group were regarded as controls, while the other three groups (10 rat per groups) were treated with ten doses of (1) free doxycycline (2.5 mg/kg), (2) DOX-SLN (2.5 mg/kg) and (3) free SLN (no antibiotics) for 10 days (once daily) [17].

After administration of the last dose at the second day, the animals were euthanized. The livers and spleens were extracted and bacterial CFUs were determined by plating the serial dilutions of organ homogenates on the TSA plates. Colonies number was determined at 4 days at 37 °C and 5% CO_2_ after incubation.

### Histopathology

To assess the in vivo efficacy of free doxycycline and DOX-SLN, spleen and liver from untreated and treated (free doxycycline, DOX-SLN and free SLN) rat were dissected out. Spleen and liver were then fixed in 10% neutral buffered formalin. After tissue processing and paraffin embedding, sections (5 µm thickness) of the tissues were prepared and processed for Hematoxylin and Eosin staining [18]. The sections were visualized using light microscopy and the inflammation and granulomatous recorded.

### Statistical analysis

To investigate the difference between the studied groups, analysis of variance (ANOVA) test was applied. The Post Hoc Tukey test was used throughout the study. All statistical tests were performed at the 0.05 level of confidence.

## Results

DOX-SLN was characterized previously [14].

### Characterization of DOX-SLN

**Fig. 1:**
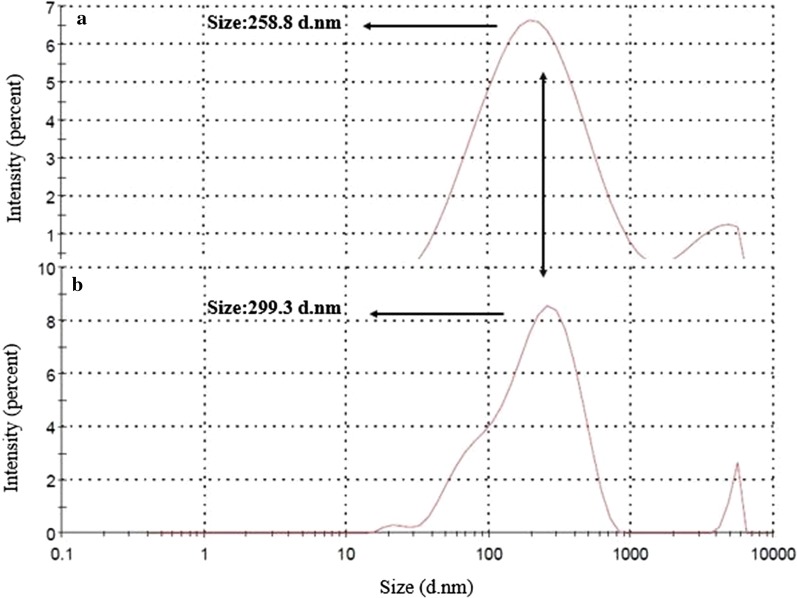
DLS size of nanoparticles; **a** SLN, **b** DOX-SLN

**Fig. 2:**
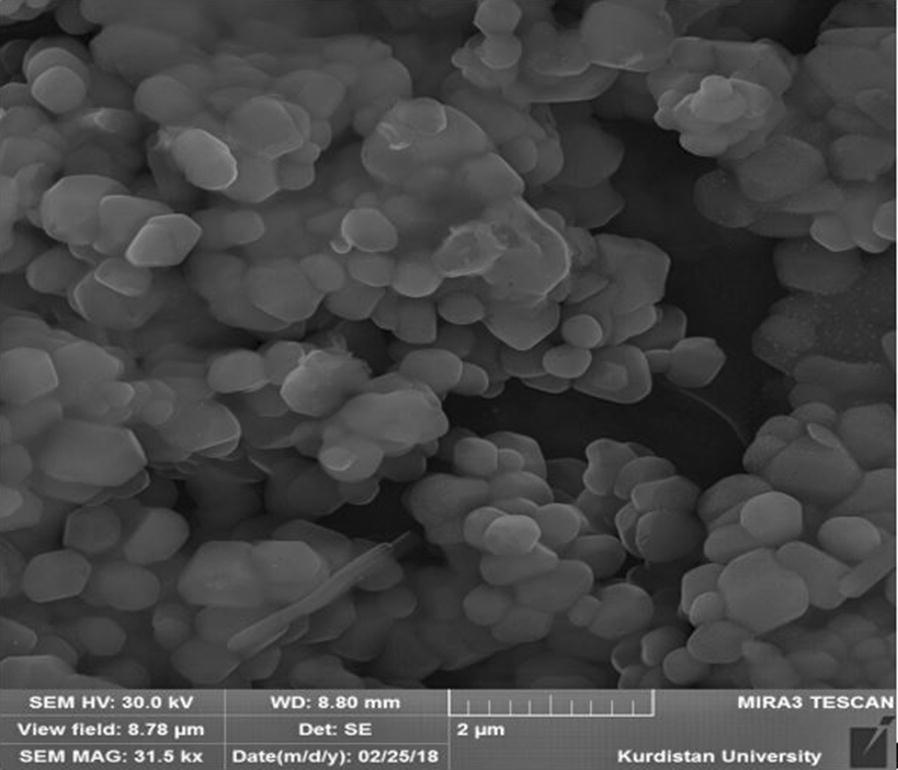
Field emission scanning electronic microscope image of DOX-SLN

DLS results of SLN shows 258.8 nm diameter size which was increased to 299.3 nm for DOX-SLN. As shown in Fig. 1, increasing the nanoparticle size from 258.8 to 299.3 nm is related to the doxycycline loading into the SLN. FE-SEM image shows the spherical DOX-SLN surface morphology with a homogeneous polydispersity (Fig. 2).

### MTT assay

**Fig. 3:**
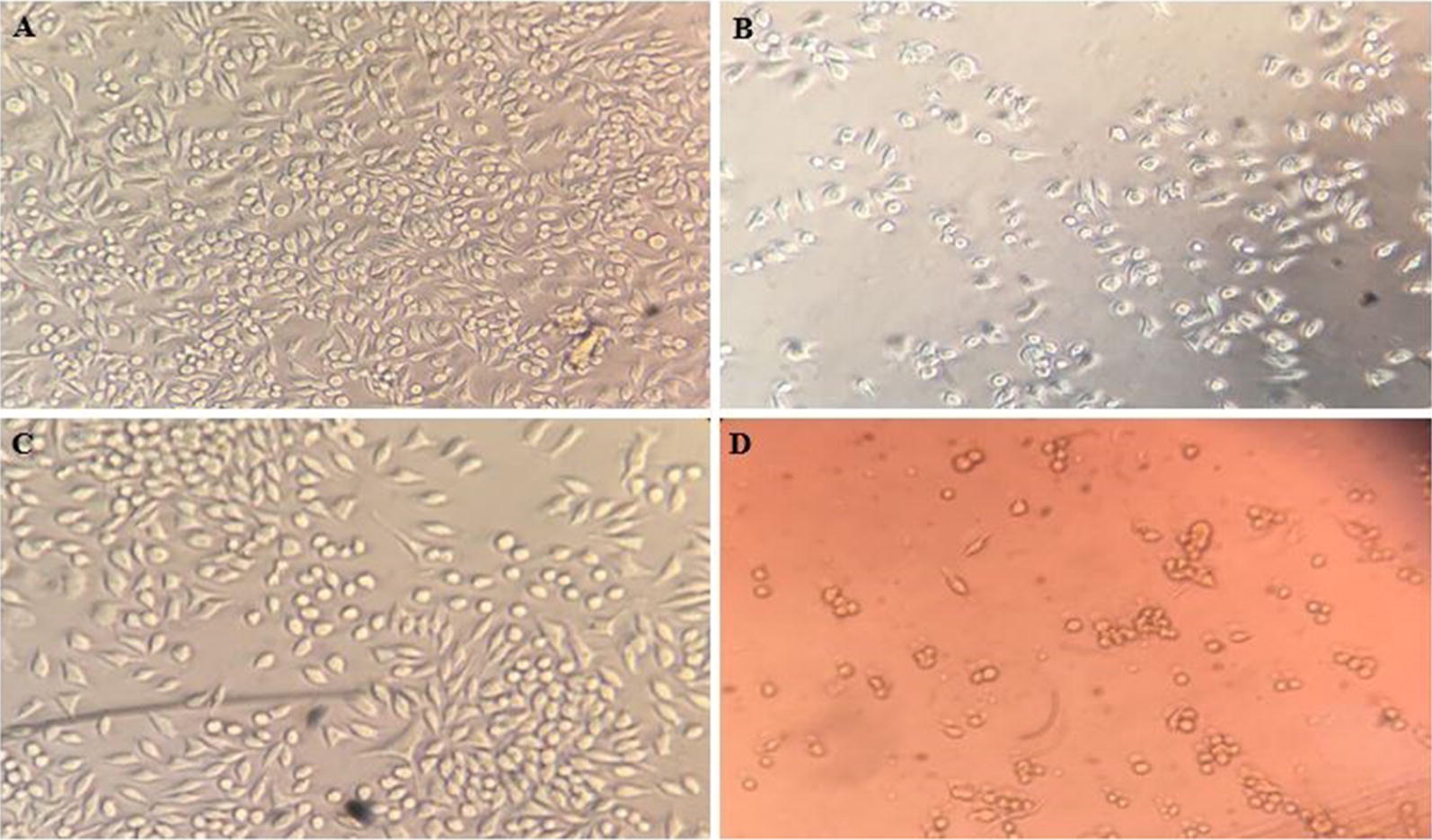
Effect of DOX-SLN and free doxycycline on J774A.1 cells after 24 h; **A** positive control: no exposure to any drug (squamous live cells attached to plate floor), **B** exposed to 3 μL DMSO (suspended dead cells disjointed from plate floor), **C** exposed to 400 μg/mL DOX-SLN, **D** exposed to 400 μg/mL free doxycycline (orange background color is because of the inherent color of doxycycline antibiotic)

To access the cytotoxicity of DOX-SLN on the J774A.1 cell line, MTT assay was used. The results of this study show that DOX-SLN has a lower toxicity that free doxycycline (Fig. 3).

### In vivo infection assay

The treatment of rat started 10 days and 5 weeks after infection with *B. melitensis* for acute and chronic stage respectively. The results are summarized in Tables 1 and 2.

**Table 1:** In vivo efficacy of DOX-SLN and free doxycycline against *B. melitensis* in acute phase

Treatment	Acute stage			
	CFUs (Log_10_) in spleen	Log CFUs reduction	CFUs (Log_10_) in liver	Log CFUs reduction
Untreated	5.03 ± 0.03	0.00	5.05 ± 0.05	0.00
Free SLN	5.02 ± 0.08	0.01	5.04 ± 0.01	0.01
DOX-SLN	4.295 ± 0.27	0.73*	4.30 ± 0.31	0.75*
Free doxycycline	4.301 ± 0.32	0.72*	4.31 ± 0.91	0.74*

Comparisons were performed between all treatments and control without treatment: * *P *< 0.05

**Table 2:** In vivo efficacy of DOX-SLN and free doxycycline against B. melitensis in chronic phase

Treatment	Chronic stage			
	CFUs (Log_10_) in spleen	Log CFUs reduction	CFUs (Log_10_) in liver	Log CFUs reduction
Untreated	4.96 ± 0.09	0.00	4.96 ± 0.01	0.00
Free SLN	4.94 ± 0.10	0.02	4.95 ± 0.12	0.01
DOX-SLN	3.51 ± 0.90	1.45*	3.55 ± 0.83	1.41*
Free doxycycline	3.67 ± 0.12	1.29*	3.68 ± 0.74	1.28*

Comparisons were performed between all treatments and control without treatment: * *P *< 0.05

In both stages, free doxycycline and DOX-SLN showed a significant (*P *< 0.05) reduction in the log CFUs of *B. melitensis* per spleen and liver compared to untreated controls in infected rat with three and ten doses of treatment.

In the acute phase, free drug and DOX-SLN equally reduced bacterial colonies from the spleen and liver of infected rats. In the chronic phase, DOX-SLN exerted a greater effect on the bacterium and reduced the bacterial colonies of the liver and spleen of rats compared to the free drug. Free SLN had no significant effect on reducing the number of *B. melitensis* colonies.

### Histopathology

**Fig. 4:**
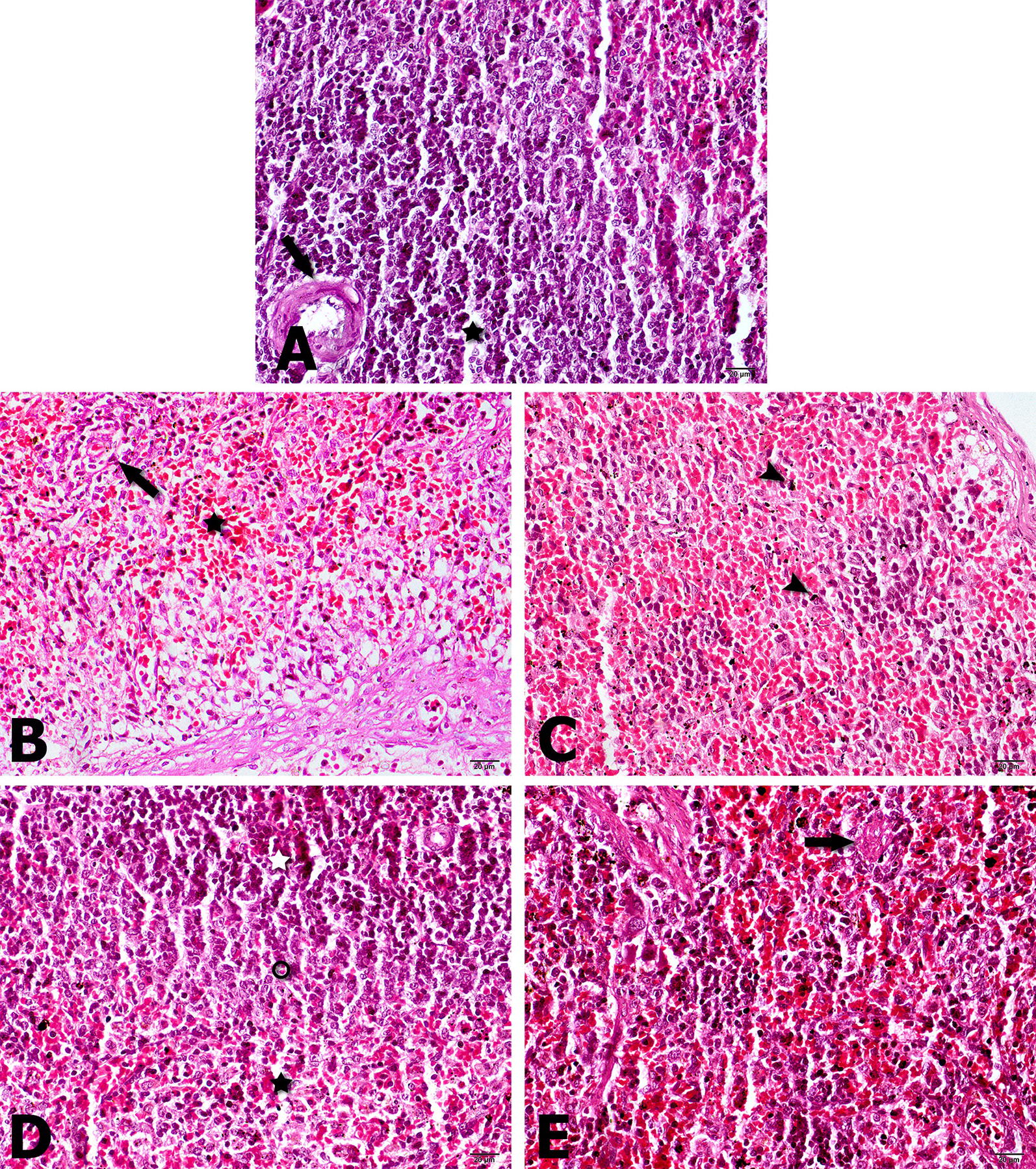
Photomicrographs of spleen; **A** Normal architecture of spleen from a healthy rat showing regular red and white pulps with central artery (arrow) and distinct germinal center (asterisk); **B** treated by free SLN: Inflammation of the organ with granulomatous reaction (arrow) and subcapsular congestion (asterisk); **C** Untreated. Diffuse inflammation and congestion of the organ with hemosiderin precipitation (arrowheads); **D** treated by DOX-SLN. Microscopic architecture of spleen is being restored with typical red pulps (black asterisk) and white pulps (white asterisk) with marginal zone (circle); **E** treated by free Doxycycline. The organ architecture is being regenerated with typical red and white pulps. However, occasional granulomatous reaction exists in the red pulp (arrow). Hematoxylin & Eosin, Magnification = ×400

**Fig. 5:**
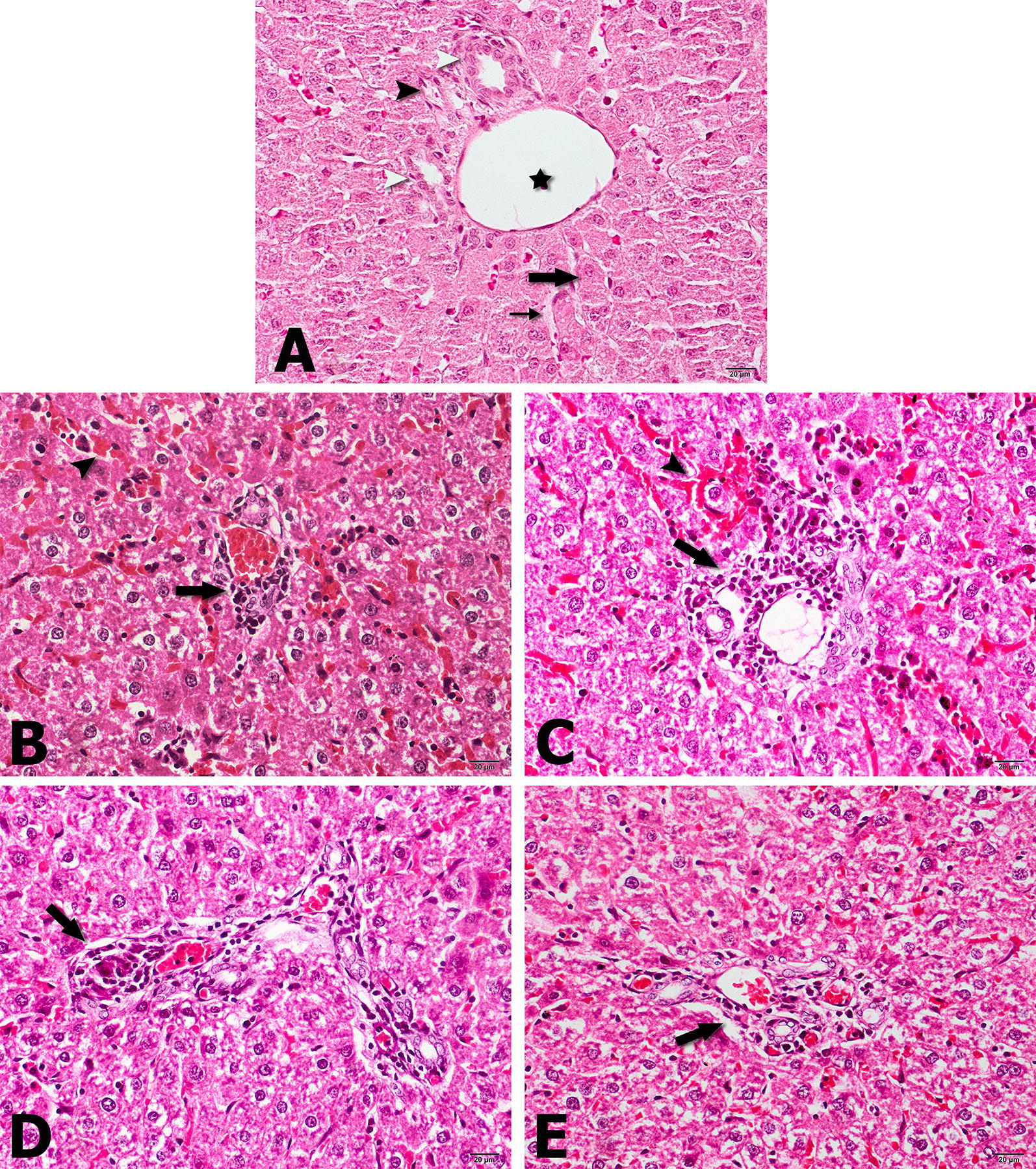
Photomicrographs of liver; **A** Normal microscopic view of the organ, with typical hepatic cords (large arrow) and sinusoids (small arrow), and characteristic portal triad showing portal venule (asterisk), hepatic arteriole (black arrowhead) and bile ductula (white arrowheads); **B** treated by Free SLN: diffuse necrosis and lipoidal degeneration of hepatocyte with foamy cytoplasm, severe sinusoidal congestion (arrowhead) and preportal infiltration of lymphocytes and macrophages (arrow); **C** untreated: diffuse necrosis and lipoidal degeneration of hepatocyte with foamy cytoplasm, severe sinusoidal congestion (arrowhead) and preportal infiltration of lymphocytes and macrophages (arrow); **D** treated by DOX-SLN: the normal architecture of liver is being restored with less preportal infiltration of inflammatory cells (arrow) and no sinusoidal congestion; **E** treated by free doxycycline: Infiltration of white blood cells (arrow) in preportal area and moderate sinusoidal congestion. Hematoxylin & Eosin, Magnification = ×400

The results of histopathological tests after treatment with DOX-SLN, free doxycycline and free SLN in the spleen and liver are shown in Figs. 4 and 5. In Fig. 4B, C, respectively, treatment with free SLN and untreated group in the spleen, and Fig. 5B, C related to treatment with free SLN and untreated group in the liver. However, inflammation and granulomatous reactions are frequently found to indicate the effect of *B. melitensis* on the spleen and liver. Figures 4D and 5D show treatment with ten doses of DOX-SLN in the spleen and liver, compared with the untreated group and normal spleen and liver tissue, with dramatic effects of healing in the spleen despite typical white and red pulp. Decreased inflammatory cells show sinus congestion in the liver.

## Discussion

The economic consequences of brucellosis and other chronic infectious diseases on societies are significant. Furthermore, the body immune system and antibiotics presented in the extracellular environment can not affect the bacteria resided inside the cell [19, 20]. Therefore, empowering drug delivery systems to penetrate the inside cell is vital to attain a higher efficacy in dealing with the intracellular infections.

**Scheme 1:**
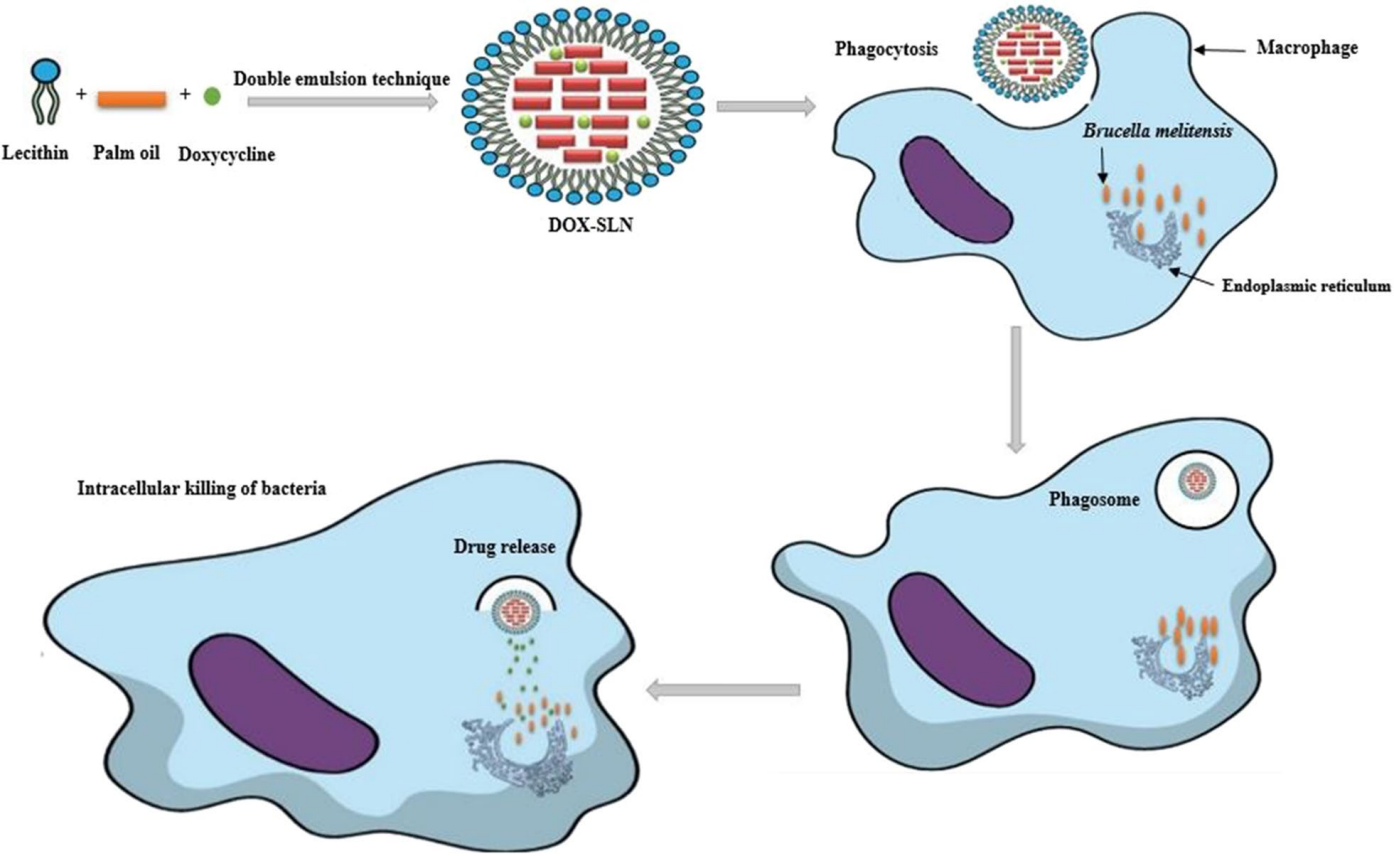
Nanoparticles formation, phagocytosis process and its effect on *B. melitensis* enclosed in macrophages

The present study endeavored to discover a new pharmaceutical strategy based on nanoscience to struggle with the chronic and recurrence forms of brucellosis. Scheme 1 shows nanoparticles formation, phagocytosis process and its effect on *B. melitensis* enclosed in macrophages.

The mean size of the optimum formulation was 299 ± 34, which is the suitable predicted size for the phagocytic activity. The results of this study are in agreement with the results of Liu et al. in which the size of the nanoparticles decreased with increasing sonication time [21]. In addition, lyophilization resulted in the increase of nanoparticle size, which is line with the findings of Chantaburanan et al. [22].

The process of involvement to Brucellosis in the experimental mouse model is divided in three phases. The first phase is the beginning of infection requiring 1 to 3 days followed by the onset of the second phase which *Brucella* replicates actively in reticuloendothelial system up to 2 weeks called the acute phase. During the third phase of infection (5–10 weeks), Brucella stabilizes in the organs and persists up to 1 year. The Antibiotic treatment challenge occurs more during chronic phase as the pathogens are not replicate actively and drug targets become limited. Most published data pointed the acute infection as the best phase to apply drug carrying nanoparticles to treat Brucellosis in mice [23].

In vivo results showed that there was a significant difference in the number of colonies of *B. melitensis* in spleen and liver in acute and chronic stages after use of DOX-SLN and free doxycycline compared to untreated group (*P *= 0.001). It was also found that the use of free SLN did not decrease in the number of bacterial colonies. In the acute stage of infection, the use of DOX-SLN and free doxycycline reduced the bacterial count of the spleen and liver almost equally (Tables 1 and 2), which may be due to the fact that the bacterium has not yet fully entered the spleen and liver macrophages and microgranule. Chronic disease is not yet established and the bacteria are directly exposed to free drug and nanoparticle. In the chronic stage, the prescription of ten doses of DOX-SLN had a significantly higher efficacy in reducing bacteria in liver and spleen than other groups (*P *< 0.001). The results are in line with those studies in which various nanoparticles have been used for the treatment [19, 24, 25]. The main difference between this study and other similar studies is that in the present study the chronic stage of treatment was investigated, while in previous studies the treatment had started only after 2 weeks of infection.

Lecaroz et al. studied the effect of gentamicin loaded in microsphere on *B. melitensis.* The results indicate the perfect impression of drug on bacterial decrement [26]. Seleem et al. made a nanoparticle using doxycycline and streptomycin in order to assess its effect on *B. melitensis* in in vivo condition [25], The results were in line with the present study in acute phase. Jain-Gupta et al. and Imbuluzqueta et al. used NPs containing gentamicin to treat infected mice in the chronic and acute phase, respectively which both results dedicated that the NPs could decrease bacteria colonies significantly from spleen and liver [19, 24].

In overall, according to the mentioned studies gentamicin has a good effect on Brucellosis treatment. Many studies used this antibiotic for encapsulation due to gentamicin disability to enter the spleen and liver macrophage cells which ultimate to the *Brucella* remarkable reduction in mice and rat. All these experiments carried out in the hope of reaching Brucellosis treatment in human despite gentamicin has well-known nephrotoxicity effect on human-like other aminoglycosides [19, 24, 27].

Jain-Gupta et al. used magnetic block ionomer complex (MBICs) for gentamicin encapsulation. After 28 doses of nanoparticle injection to the rats infected to *B. melitensis*, they assessed the nephrotoxic effect of the drug in a histopathology study. No difference was observed after neither encapsulated gentamicin nor free drug. The main point is that relapse would occur when the patient has entered the chronic stage which was the main focus of the present study. Liver and spleen tissues were histologically investigated by two pathologists after treatment with ten doses and the results were quite promising [19].

According to Fig. 4B, in spleen tissue treated with free SLN, inflammation with granulomatous reaction and subscapular congestion was seen. In untreated rats, diffuse inflammation and congestion of the organ with hemosiderin precipitation was observed, which pointed the severe effect of *Brucella* on spleen tissue. The spleen of rats treated with DOX-SLN, is being restored with typical red pulps and white pulps with marginal zone. This is due to the fact that nanoparticle has no toxic effect on the tissue, but after ten doses of nanoparticle injection it has a great therapeutic effect. Also DOX-SLN has better therapeutic effect than free doxycycline, because granulomatous reaction with red pulps is seen after doxycycline usage. In histopathology study on the liver of infected, but untreated rats, the liver tissue has diffuse necrosis and lipoidal degeneration of hepatocyte with foamy cytoplasm, severe sinusoidal congestion and preportal infiltration of lymphocytes and macrophages, which shows the effects of brucellosis on liver tissue. The liver tissue of rats treated with DOX-SLN is being restored with less preportal infiltration of inflammatory cells with no sinusoidal congestion. Generally, after treatment with DOX-SLN, spleen and liver tissues were successfully improved compared to those tissues extracted from untreated rats. Moreover, there were no granulomatous reaction after treatment of these tissues, demonstrating the effectiveness of nanoparticles in killing bacteria presented in the macrophages of these tissues.

## Conclusion

The double emulsion method is appropriate to encapsulation of doxycycline which is a hydrophilic drug so that PDI, zeta potential and particle size were favorable for our aims. The results of this study demonstrated that DOX-SLN is more effective than free doxycycline in dealing with *B. melitensis* phagocyted by the macrophages. This is because of the slow and continuous release of drug without any cytotoxic effects. This study indicated the potential of SLN for delivering sustained therapeutic antibiotics concentrations in the liver and the spleen, the target organs for intracellular infections such as brucellosis. According to the above, to treat such disease caused by intracellular bacteria like *B. melitensis* with the high rate of recurrence, nanoparticles can be considered as a promising tool.

## Limitations

The results of this study showed that using DOX-SLN is more effective than free doxycycline in dealing with *B. melitensis* phagocyted by the macrophages, if using multiple-drug combination that are routinely used to treat brucellosis, could be more effectively and also utilizing other nanoparticles and comparing its effect on *B. melitensis*.

## Data Availability

The data can be accessible to the interested researchers by the corresponding authors on reasonable request.

## Abbreviations

SLN: solid lipid nanoparticles
DOX-SLN: doxycycline-loaded solid lipid nanoparticles
PDI: polydispersity index
NPs: nanoparticles
FTIR: Fourier-transform infrared spectroscopy
DSC: differential scanning calorimetry
WHO: World Health Organization
DLS: dynamic light scattering
FE-SEM: field emission scanning electronic microscope
HPLC: high-performance liquid chromatography
TSB: tryptic soy broth
TSA: tryptic soy agar
FBS: fetal bovine serum
DMEM: Dulbecco’s Modified Eagle’s media
MTT: 3-(4,5-dimethylthiazol-2-yl)-2,5-diphenyltetrazolium bromide
MIC: minimum inhibitory concentration
ANOVA: analysis of variance
